# Origins, evolution, and physiological implications of de novo genes in yeast

**DOI:** 10.1002/yea.3810

**Published:** 2022-08-24

**Authors:** Saurin B. Parikh, Carly Houghton, S. Branden Van Oss, Aaron Wacholder, Anne‐Ruxandra Carvunis

**Affiliations:** ^1^ Department of Computational and Systems Biology, School of Medicine, Pittsburgh Center for Evolutionary Biology and Evolution University of Pittsburgh Pittsburgh Pennsylvania USA

**Keywords:** de novo genes, evolutionary biology, genome biology, smORFs, systems biology

## Abstract

De novo gene birth is the process by which new genes emerge in sequences that were previously noncoding. Over the past decade, researchers have taken advantage of the power of yeast as a model and a tool to study the evolutionary mechanisms and physiological implications of de novo gene birth. We summarize the mechanisms that have been proposed to explicate how noncoding sequences can become protein‐coding genes, highlighting the discovery of pervasive translation of the yeast transcriptome and its presumed impact on evolutionary innovation. We summarize current best practices for the identification and characterization of de novo genes. Crucially, we explain that the field is still in its nascency, with the physiological roles of most young yeast de novo genes identified thus far still utterly unknown. We hope this review inspires researchers to investigate the true contribution of de novo gene birth to cellular physiology and phenotypic diversity across yeast strains and species.

## Take Aways

- Several yeast genes of recent de novo origin play important cellular roles.
- Yeasts express thousands of de novo sequences with unknown biology.
- Yeasts are well suited to address fundamental questions about de novo gene birth.

## Graphical abstract

This paper makes a case for why yeasts are particularly well suited to address fundamental questions about de novo gene emergence. Several yeast genes of recent de novo origin are known to play important cellular roles. Yeasts also express thousands of species‐specific and strain‐specific de novo open reading frames with unknown biology that offer exciting avenues for discovery.

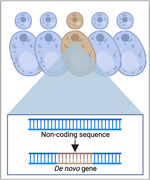

## 1 A BRIEF HISTORY OF DE NOVO GENE BIRTH RESEARCH

De novo gene birth is the process by which new genes evolve from sequences that were previously noncoding (Tautz, 2014; Tautz & Domazet‐Loso, 2011; Van Oss & Carvunis, 2019). Once thought to be exceedingly rare (Jacob, 1977), de novo gene birth has now been observed in a wide variety of taxa (Baalsrud et al., 2018; Cai et al., 2008; Chen et al., 2010; Heinen et al., 2009; Khan et al., 2020; Li et al., 2010; Reinhardt et al., 2013; Weisman, 2022; Xie et al., 2019). Several studies have described how young de novo genes that exist in only a single species can play important biological roles through species‐specific molecular mechanisms (Bungard et al., 2017; Cai et al., 2008; Li et al., 2010, 2014; Xie et al., 2019; Zhuang et al., 2019). The process of de novo gene birth has therefore received considerable recent attention as a major potential source of genetic, structural, and phenotypic novelty (Abrusan, 2013; Bornberg‐Bauer et al., 2021; Capra et al., 2010; Chen et al., 2013; Knopp et al., 2019; Lee et al., 2019; McLysaght & Guerzoni, 2015; McLysaght & Hurst, 2016; Reinhardt et al., 2013; Schlotterer, 2015).

Yeasts have played a central role in the field of de novo gene birth since its inception. When the *Saccharomyces cerevisiae* genome was sequenced in 1996, approximately 6000 open reading frames (ORFs) longer than 300 nucleotides were predicted to be protein‐coding genes (Goffeau et al., 1996). Of these, around 30% lacked identifiable homologs among known genes from other species—that is, they were “orphan genes” (Dujon, 1996). The sequencing of additional genomes over the course of the subsequent decades led to the identification of homologs for many of these orphans (Brachat et al., 2003; Cliften et al., 2003; Kellis et al., 2003; Riley et al., 2016; Shen et al., 2018; Weisman et al., 2020). Nevertheless, several hundred remained homolog‐free, unable to be grouped into any gene family. Since de novo gene birth was considered highly implausible, such lack of cross‐species conservation combined with the absence of experimental evidence was thought to indicate a lack of function. The remaining orphans were therefore initially presumed to correspond to mis‐annotations, unlikely to encode functional protein‐coding genes, and relegated to the status of “dubious” ORFs (Fisk et al., 2006). However, a 2008 survey of dubious ORFs showed that most were in fact expressed and detected in high‐throughput functional genomics assays, suggesting that they did not correspond to mere mis‐annotations but may encode *bona fide* orphan genes (Q. R. Li et al., 2008). The same year, Cai et al. (2008) demonstrated that the *S. cerevisiae* orphan gene *BSC4* was of de novo origin.

*BSC4* was originally identified as a translated ORF exhibiting Sup35‐dependent translational readthrough (Namy et al., 2003). Cai et al. (2008) then showed that *BSC4* evolved recently in the *S. cerevisiae* lineage via point mutations in a locus that was previously noncoding. This was the first demonstration that a full‐length protein‐coding gene can emerge de novo in any species. The authors showed that *BSC4* increases in expression level throughout the stationary phase and, based on synthetic lethal interactions with *RPN4* and *DUN1*, proposed that Bsc4 is involved in DNA repair during the stationary phase to enable the transition from nutrient‐rich to nutrient‐poor environments. A decade after this initial characterization, Bsc4 became the first protein encoded by a de novo gene to be structurally characterized in any species. Unlike typical conserved yeast proteins, it was found to exhibit a rudimentary “molten globule” fold with high beta‐sheet content and a hydrophobic core (Bungard et al., 2017).

**Figure 1:**
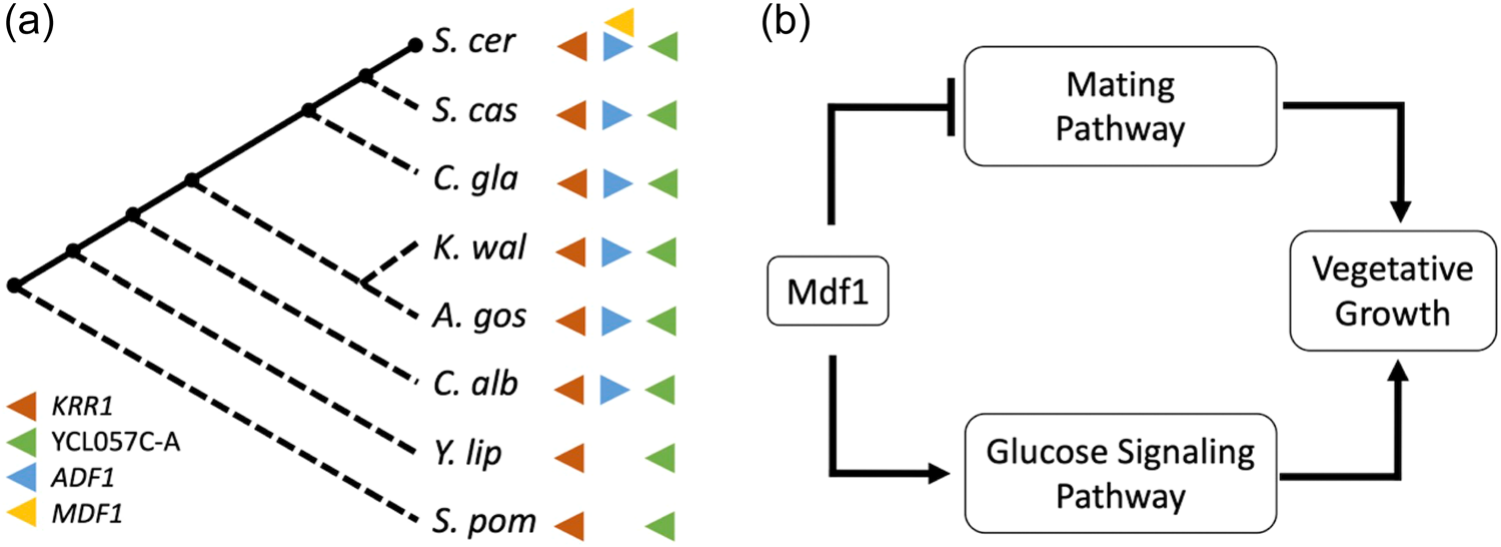
*MDF1*: A de novo‐evolved gene that integrates into essential biological pathways. (a) Phylogeny‐ and synteny‐based analysis of various fungi revealed that *MDF1* emerged specifically in *S. cer* subsequent to its split from *S. cas*. At the same time, *ADF1*, an antisense gene to MDF1, is conserved in all but the most distant member of the hemiascomycete subdivision of fungi. The *MDF1* syntenic block is shown to the right of the phylogenetic tree. (Li et al., 2010) (b) Mdf1 promotes vegetative growth by suppressing the mating pathway and enhancing the glucose signaling pathway (Li et al., 2014). *A. gos*, *Ashbya gossypii*; *C. alb*, *Candida albicans*; *C. gla*, *Candida glabrata*; *S. cas*, *Saccharomyces castellii*; *S. cer*, *Saccharomyces cerevisiae*; *S. pom*, *Schizosaccharomyces pombe*; *Y. lip*, *Yarrowia lipolytica*.

Shortly following the characterization of *BSC4*, Li et al. (2010, 2014) deployed an exhaustive set of experiments and analyses (Table 1) to generate the most complete characterization of a yeast de novo gene to date. Their studies showed that the *MDF1* ORF emerged de novo in *S. cerevisiae* in the previously noncoding sequence antisense to a conserved protein‐coding gene, *ADF1* (Figure 1a). Interestingly, Adf1 represses transcription of *MDF1* by binding to its promoter such that sense and antisense expression at this locus have antagonistic physiological effects. When expressed, Mdf1 promotes fermentation and suppresses mating by physically interacting with Snf1 and Matα2 (Figure 1b). In other words, the young de novo gene *MDF1* mediates the crosstalk between reproduction and vegetative growth through a *S. cerevisiae‐*specific molecular mechanism. The case of *MDF1* illustrates how, contrary to prior assumptions, young ORFs that emerged de novo in noncoding sequences and lack cross‐species conservation can encode proteins with key cellular roles.

**Table 1:** Applying the evolutionary systems biology approach to the investigation of *MDF1*

Categories	Test for evidence	Results
Sequence	Comparative genomics	It is under positive selection.
	PSI‐BLAST	There are no significantly homologous ORFs in all of the other organisms examined beyond two short, truncated ORFs in the close relatives *Saccharomyces bayanus* and *Saccharomyces mikatae*.
	Synteny	The intergenic region between flanking genes could not encode a protein in other species due to the presence of multiple stop codons.
Expression	Strand‐specific RT‐PCR	*MDF1* is only expressed in *Saccharomyces cerevisiae*.
	Western blot	Positive signal for the protein.
Structure	Structure prediction server—PORTER	Mdf1 mimics Mata1 in having a three‐helix‐domain that can bind to Mat*α*2.
Localization	Fluorescent tagging	Mdf1 exists in the cytoplasm and nucleus.
Interaction and mechanism	Chromatin immunoprecipitation	Adf1 binds to the upstream region of *MDF1*.
		Mdf1 binds haploid‐specific genes (*MATα1*, *STE4*, *STE5*, *FUS1*, *FUS2*, *FUS3*, *GPA1*, *SST2*, and *RME1*).
	Gel electrophoresis	*ATP1*, *PGK1*, *MDH1*, and *SAM1* expression is increased in *MDF1 ADF1Δ* strains.
	Microarray	Downregulation of mating pathway (MAPK).
	Semi‐quantitative RT‐PCR	MAPK pathway genes (*STE3*, *STE12*, *FUS1*, *FUS3*) are downregulated.
	Complementation assay	Overexpression of *MATα1* gene rescues the mating ability of an *mdf1Δ* mutant.
	Yeast two‐hybrid assay	Mdf1 interacts with Matα2.
	Pull‐down assay	Mdf1 interacts with Matα2.
	Electrophoretic mobility shift assays	Mdf1 and Matα2 are bound to each other and function in a mutually dependent manner.
Phenotype and fitness	Competition experiment	*MDF1 ADF1Δ* strain grows more quickly than the wild‐type strain.
	Growth rate analyses	
	Mating assay	*MDF1 ADF1Δ* is less successful at mating. No such effect is seen in closely related species.

Abbreviations: MAPK, mitogen‑activated protein kinase; ORF, open reading frame; RT‐PCR, reverse transcription polymerase chain reaction.

## 2 METHODS FOR INFERRING DE NOVO ORIGIN

**Figure 2:**
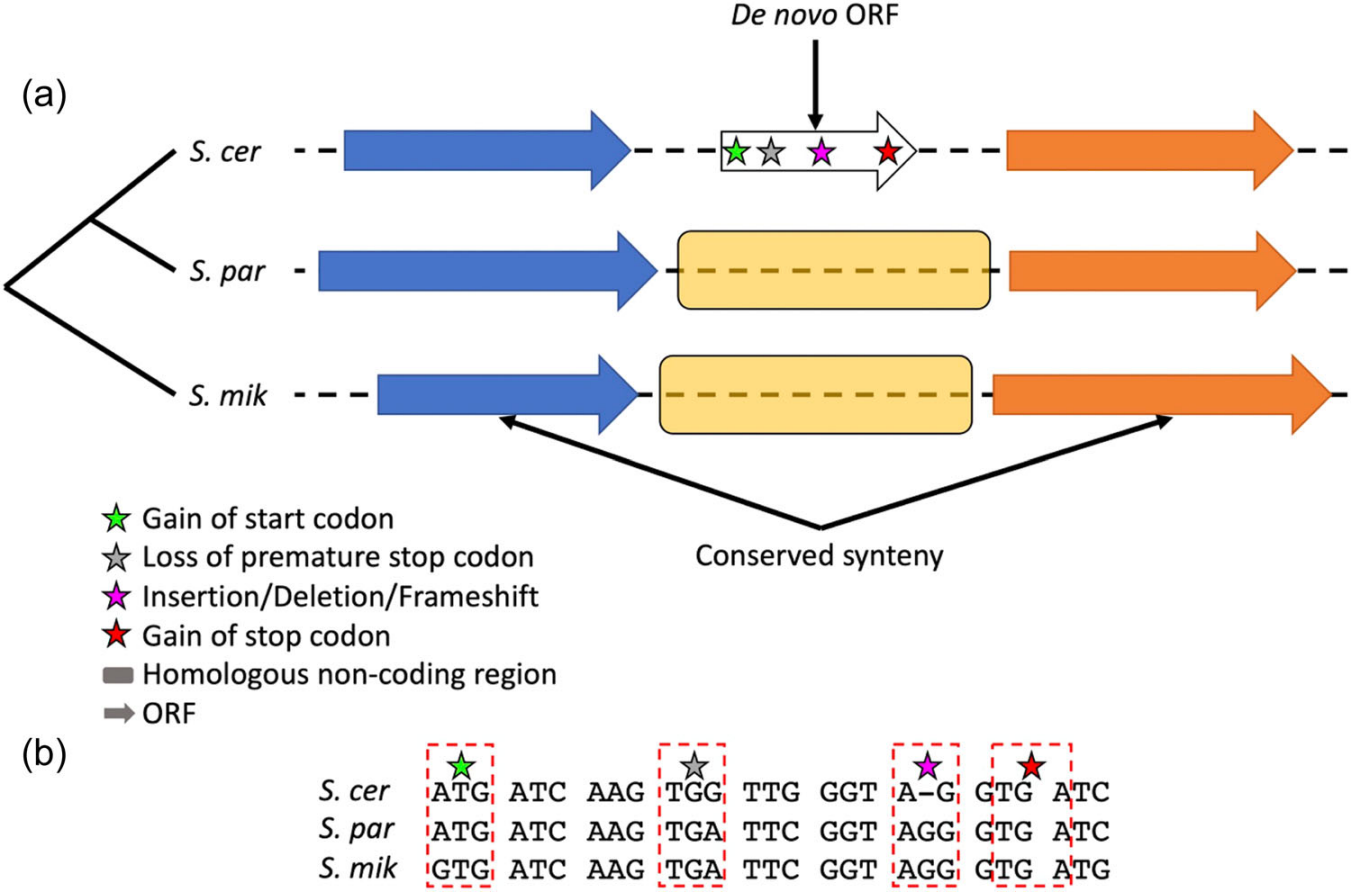
Pictographic representation of a hypothetical de novo ORF in *Saccharomyces cerevisiae*. (a) A combination of conserved synteny and phylostratigraphy is used to identify the homologous region of interest (highlighted in yellow) in the closely related species. This region of interest can be used to identify enabling mutations across the lineage that led to the de novo ORF in the focal species (*S. cerevisiae* in this case). The enabling mutations can include but are not limited to a gain of the start codon (green star), loss of premature stop codon (gray star), insertion–deletion and/or a frameshift (pink star) and a gain of stop codon (red star). Figure inspired by Vakirlis and McLysaght (2019). (b) A hypothetical example of enabling mutations that occurred along the lineage to result in a de novo ORF in the focal genome. Changes highlighted within boxes are possible enablers. Identification of one or more of such mutations (example gain of the start codon) are needed to provide convincing evidence of de novo ORF emergence. ORF, open reading frame; *S. cer*, *Saccharomyces cerevisiae*; *S. mik, Saccharomyces mikatae*; *S. par, Saccharomyces paradoxus*.

The most convincing evidence that an ORF originated de novo is the identification of a set of one or more “enabling mutations” that arose in previously noncoding sequences within the lineage resulting in a new ORF (e.g., mutation/s creating a new start codon) (McLysaght & Hurst, 2016). This is done by aligning the locus containing the ORF of interest with syntenic orthologous DNA regions in closely related species and showing that the enabling mutations are absent in these species, that is, showing that the orthologous DNA regions are truly noncoding (Vakirlis & McLysaght, 2019; Figure 2). A study applying this approach confirmed the de novo origin for 30 *Saccharomyces* ORFs (Vakirlis et al., 2018).

Such synteny analyses for de novo gene birth inference can be further refined using ancestral sequence reconstruction approaches. This was demonstrated for the first time in any species with the de novo *S. cerevisiae* ORF YBR196C‐A (Vakirlis, Acar, et al., 2020). Ancestral reconstruction at this locus showed not only how enabling mutations conferred coding potential to an ancestrally noncoding DNA region, but also how subsequent frameshifts and substitutions have led to the rapid evolution of the initial ORF, leading to loss in some lineages and substantial changes in length and primary sequence in others. These mutational processes led to the emergence of a small species‐specific transmembrane protein in *S. cerevisiae* that localizes at the endoplasmic reticulum and promotes larger colony growth when overexpressed. A subsequent study (Papadopoulos et al., 2021) used ancestral reconstruction to retrace the evolutionary history of 70 candidate de novo genes identified by previous studies (Carvunis et al., 2012; Lu et al., 2017; Vakirlis et al., 2018; Wu & Knudson, 2018), and reported that most de novo enabling mutations corresponded to frameshifts and loss‐of‐stop events leading to the merging of two small intergenic ORFs.

This complexity can obfuscate the identification of enabling mutations from syntenic alignment alone when one aims to use automated sequence analyses. To circumvent this challenge, a recent study (Wacholder et al., 2021) adapted a Reading Frame Conservation metric (Kellis et al., 2003) calculated from syntenic alignments to identify all ORFs in the *S. cerevisiae* genome with noncoding orthologous regions in other *Saccharomyces* species. These ORFs were then classified into candidate pseudogenes when distant homologs could be identified through sequence similarity searches in the fungal lineage, or candidate de novo ORFs when the corresponding protein sequence was lineage‐specific. This analysis estimated that 251 annotated *S. cerevisiae* ORFs (7 verified, 96 uncharacterized, 148 dubious) emerged de novo.

While approaches based on synteny and enabling mutations are now considered standard, phylostratigraphy‐based approaches have been widely used in earlier studies of gene birth. In phylostratigraphy, the origin of a new gene is inferred in the most recent common ancestor of all species with a homolog identified by sequence similarity searches (Domazet‐Loso et al., 2007). Three groups have performed phylostratigraphy analyses on the *S. cerevisiae* genome, providing lists of hundreds of *S. cerevisiae* orphan genes (Carvunis et al., 2012; Lu et al., 2017; Vakirlis et al., 2018); such analyses have also been conducted on *Lachancea* yeasts (Vakirlis et al., 2016). However, these results must be interpreted with caution because orphan genes can originate via several evolutionary mechanisms other than de novo gene birth, including lateral transfer and duplication followed by extreme sequence divergence (Long et al., 2003; Van Oss & Carvunis, 2019). When an orphan gene identified by phylostratigraphy is present in at least two taxa, it is possible to estimate the likelihood that it has acquired a unique sequence through extreme sequence divergence by extrapolating an estimate of its evolutionary rate (Weisman et al., 2020). However, when a gene is found only in one species with no detectable homolog at all, analyses of synteny and enabling mutations are required to infer the mechanism of origin. A recent analysis (Vakirlis, Carvunis, et al., 2020) showed that sequence divergence is not the main source of orphan genes in *S. cerevisiae*, suggesting that de novo gene birth may play a major role in generating molecular novelty.

A fundamentally different strategy for de novo gene birth inference consists in comparing genome expression patterns, rather than ORF sequences, between yeast strains and species. Indeed, de novo gene birth can take place when a pre‐existing noncoding RNA acquires a novel ORF and becomes translated (“transcription first”), or when a pre‐existing ORF becomes transcribed and translated (“ORF first”) (Schlotterer, 2015). In this latter case, the “enabling mutations” would be those that lead to a novel transcription or translation event rather than those that lead to a novel ORF. These are harder to identify by DNA sequence analysis than mutations enabling the emergence of an ORF, as the genetic determinants of expression changes over evolutionary time are not as well understood. It is however well established that the yeast lineage undergoes substantial evolutionary transcriptional turnover (H. Li & Johnson, 2010). Lu et al. (2017) identified 4340 putative *S. cerevisiae*‐specific de novo genes that are transcribed but share no orthologues in other *Saccharomycetaceae*, most of which were inferred to have arisen from transcript isoforms of ancient genes. By comparing the transcriptomes of *S. cerevisiae* and 10 other yeast species, Blevins et al. (2021) identified 213 de novo‐originated transcripts in *S. cerevisiae*, half of which were in the antisense orientation of other genes and many of which appeared to be translated. At a finer evolutionary scale, Durand et al. (2019) analyzed the turnover of ribosome‐associated transcripts among wild *Saccharomyces paradoxus* strains and identified 447 lineage‐specific translation events (Durand et al., 2019). While most were attributable to lineage‐specific ORF gains and losses, several instances appeared to have been potentiated by lineage‐specific increases in expression level.

The prevalence of de novo gene birth in yeast is supported by overwhelming comparative genomic evidence from the aforementioned studies. Yet, there is currently no definitive, community‐approved list of which yeast genes have originated de novo. Different approaches yield different—though overlapping—results (Blevins et al., 2021; Papadopoulos et al., 2021). The issue lies, in part, in that there is no consensus operational definition of what constitutes a “gene” in the context of de novo gene birth, where the signatures of evolutionary conservation typically relied on to predict functionality are absent (Keeling et al., 2019). Further developments of computational methods for the detection of de novo‐originated genes are also much needed for the advancement of the field (Li et al., 2022). Such advances are more challenging to attain in the yeast lineage than in other eukaryotic lineages whose genomes tend to evolve more slowly. Yet, as a plethora of yeast genomes have now been sequenced (Kurtzman et al., 2011; Peter et al., 2018; Shen et al., 2018; Vakirlis et al., 2016), exciting opportunities for large‐scale comparative and evolutionary studies of de novo gene emergence in yeasts are arising.

The increasing availability of intraspecies genome sequences in yeast has also revealed substantial genetic diversity, distinguishing between the “core” and the “accessory” genomes, the latter containing genes specific to sets of isolates or individual strains (McCarthy & Fitzpatrick, 2019). Exploring the extent to which the accessory genome is comprised of de novo genes will likely shed light on the mechanisms by which rapid genetic evolution mediates rapid phenotypic and ecological adaptation. Along these lines, a recent study found that only 41% of young de novo ORFs identified in the S288C reference annotation were fixed in the *S. cerevisiae* species, while most were still segregating (Vakirlis, Acar, et al., 2020). Future studies integrating evolutionary dynamics of sequence and expression variation at the population level will be instrumental in deriving models of ORF and transcript evolution in real time, to shed light on the full extent of de novo gene emergence and its impacts on the diversity of the yeast lineage.

## 3 THE “NONCANONICAL TRANSLATOME” AS A RESERVOIR FOR DE NOVO GENE BIRTH

The first unbiased genome‐scale transcriptomic studies reported that most of the *S. cerevisiae* genome is transcribed (David et al., 2006; Nagalakshmi et al., 2008). Soon after, the first ribosome profiling studies revealed widespread translation outside of annotated *S. cerevisiae* genes (Brar et al., 2012; Carvunis et al., 2012; Ingolia et al., 2009; Wilson & Masel, 2011). Shortly following these discoveries in yeast, the phenomenon was also reported in other taxa spanning the tree of life (Ruiz‐Orera & Alba, 2019). All these “noncanonical” translated elements had been missed by genome annotations because they tend to be short and rapidly evolving. The “translatome” is much larger, and much more diverse, than currently reflected in genome annotation databases.

Noncanonical translation was originally predicted by early models of de novo gene birth that were largely built on data from yeast (Cai et al., 2008; Carvunis et al., 2012; Masel, 2006; Wilson & Masel, 2011). These models postulated that some of the hundreds of thousands of short ORFs that appear and disappear continuously during the evolution of noncoding sequences could, if transcribed, become translated and expose new genetic variation to the action of natural selection. Those “proto‐genes” (Carvunis et al., 2012) with deleterious translation products would be purged away, while those with nearly neutral or adaptive effects would constitute a reservoir for de novo gene emergence. Multiple studies have now uncovered that many, if not most, noncanonical translated elements in yeast are of de novo origin (Durand et al., 2019; Spealman et al., 2018; Wacholder et al., 2021). Most recently, Wacholder et al. (2021) combined Ribo‐seq data from 42 published studies and identified strong translation evidence for almost 20,000 noncanonical *S. cerevisiae* ORFs, including 12,129 of apparent de novo origin based on Reading Frame Conservation analyses (Wacholder et al., 2021).

Future empirical studies are needed to estimate the true size of the yeast translatome, considering the expanding genetic diversity of the yeast genome and pan‐genome. Computational predictions are not yet possible, as the molecular signals governing which noncanonical ORFs are translated in vivo remain unknown. A small number of proteomic and microscopy studies have detected the protein products of some of these noncanonical translation events in yeast cells (He et al., 2018; Lu et al., 2017; Yagoub et al., 2015), but the vast majority remain undetected. The de novo‐translated ORFs include upstream ORFs and downstream ORFs (translated ORFs located upstream and downstream of annotated coding sequences, respectively) as well as ORFs translated from transcripts containing no annotated gene (Blevins et al., 2021; Carvunis et al., 2012; Durand et al., 2019; Li et al., 2021; Smith et al., 2014; Wacholder et al., 2021; Wilson & Masel, 2011). To what extent the noncanonical translatome generates an entirely novel proteome or yields rapidly degraded products that serve to regulate translation and transcript stability remains an open research question. Unknown too is the proportion of the noncanonical translatome that corresponds to translation “noise” and does not contribute to fitness. The fraction of de novo emerged noncanonical translated ORFs that become fixed into de novo genes maintained by selection is estimated to be low (Carvunis et al., 2012; Vakirlis et al., 2018; Wacholder et al., 2021). The fact that cells exert a considerable amount of energy to translate so many novel ORFs raises the question of whether such pervasive translation confers an adaptive fitness advantage, beyond providing the raw material for gene birth.

## 4 INSIGHTS INTO THE FEATURES OF DE NOVO GENES AND MECHANISMS OF DE NOVO EMERGENCE

Yeast de novo ORFs, whether annotated or not, tend to share general characteristics: Their primary sequences tend to be very similar to those of intergenic ORFs, and they tend to be short, rapidly evolving, and often expressed in both lineage‐specific and condition‐specific manners (Basile et al., 2017; Blevins et al., 2021; Carvunis et al., 2012; Durand et al., 2019; Ekman & Elofsson, 2010; Li et al., 2021; Papadopoulos et al., 2021; Vakirlis et al., 2018; Wu & Knudson, 2018). These characteristics are thought to derive directly from their de novo emergence and to be associated with possible physiological corollaries. For example, condition‐specific expression of yeast de novo ORFs has been reported in the context of various stresses (Blevins et al., 2021; Carvunis et al., 2012; Doughty et al., 2020; Li et al., 2021; Wacholder et al., 2021). Could these species‐specific translated elements represent a rapidly evolving part of the cell's response to stress?

Vakirlis, Acar et al. (2020) provided some evidence to this question by showing that overexpression of young *S. cerevisiae* de novo ORFs with predicted transmembrane domains can increase colony growth under nitrogen or carbon limitation. Transmembrane domains are overrepresented among annotated de novo ORFs in *S. cerevisiae* (Carvunis et al., 2012; Vakirlis, Acar, et al., 2020), but the cellular mechanisms by which increased expression of species‐specific transmembrane domains would allow cells to adapt to starvation stress remain to be elucidated. Interestingly, Vakirlis, Acar et al. (2020) did elucidate the evolutionary mechanisms giving rise to the de novo origination of ORFs with transmembrane domains as a direct result of codon biases in the genetic code, whereby transmembrane residues tend to be encoded by thymine‐rich codons. A “transmembrane‐first” model was therefore proposed whereby translation of intergenic sequences that are rich in thymine have a high propensity to generate transmembrane peptides, which in turn are more likely to be adaptive and retained by natural selection. The transmembrane‐first model is, to date, the only proposed model that directly ties molecular mechanisms of de novo gene birth to a specific biophysical protein property associated with an adaptive fitness advantage.

Several studies, however, have identified additional properties of yeast de novo ORFs that are also linked to their evolutionary trajectories and possibly to their physiological roles. In particular, the specific genomic location where de novo emergence takes place appears to greatly influence primary sequence, transcriptional regulation, and evolutionary rate. Vakirlis et al. (2018) reported a strong over‐representation of de novo ORFs at GC‐rich loci across multiple yeast lineages. These loci are depleted in stop codons and often correspond to divergent gene promoters, suggesting a regulatory relationship between these de novo ORFs and their conserved neighbors. Blevins et al. (2021) identified many de novo transcripts located on the opposite strand of conserved genes and coregulated with their overlapping counterparts in response to stress. Loci opposite protein‐coding genes also tend to be depleted in stop codons. An over‐representation of yeast de novo ORFs has been reported in rapidly evolving subtelomeric regions (Carvunis et al., 2012) and recombination hot spots (Vakirlis et al., 2018). It is tempting to speculate that genomic regions that are transcriptionally active, fast‐evolving, or depleted in stop codons favor not only the emergence but also the functional evolution and retention of de novo genes.

Collectively, these studies suggest the existence of diverse evolutionary avenues for de novo gene birth, each possibly associated with different biophysical protein properties and phenotypic impacts. It is unclear how young de novo ORFs change over time, although some evidence suggests a trend towards increasing foldability (Papadopoulos et al., 2021). It may be that distinct selective pressures favor the emergence of distinct types of proteins in different environments. For example, while intrinsic disorder is predicted to be rare among yeast de novo genes in general (Basile et al., 2017; Carvunis et al., 2012; Ekman & Elofsson, 2010; Vakirlis et al., 2018; Vakirlis, Acar, et al., 2020), it is observed in high excess in older de novo genes from the *Lachancea* lineage (Vakirlis et al., 2018). It is thought that de novo genes increase in length, expression level, and cellular interactivity over time (Abrusan, 2013; Carvunis et al., 2012; Lu et al., 2017; Tautz, 2014), but more mechanistic research is needed to fully understand the long‐term evolutionary dynamics of de novo gene origination and evolution. The physiological implications of de novo gene emergence are in dire need of further study as well. No noncanonical de novo‐translated ORFs, and very few annotated de novo genes, have been deeply characterized to date.

## 5 PROPOSED EVOLUTIONARY SYSTEMS BIOLOGY FRAMEWORK FOR FUTURE INVESTIGATIONS OF DE NOVO GENE BIRTH IN YEAST

**Figure 3:**
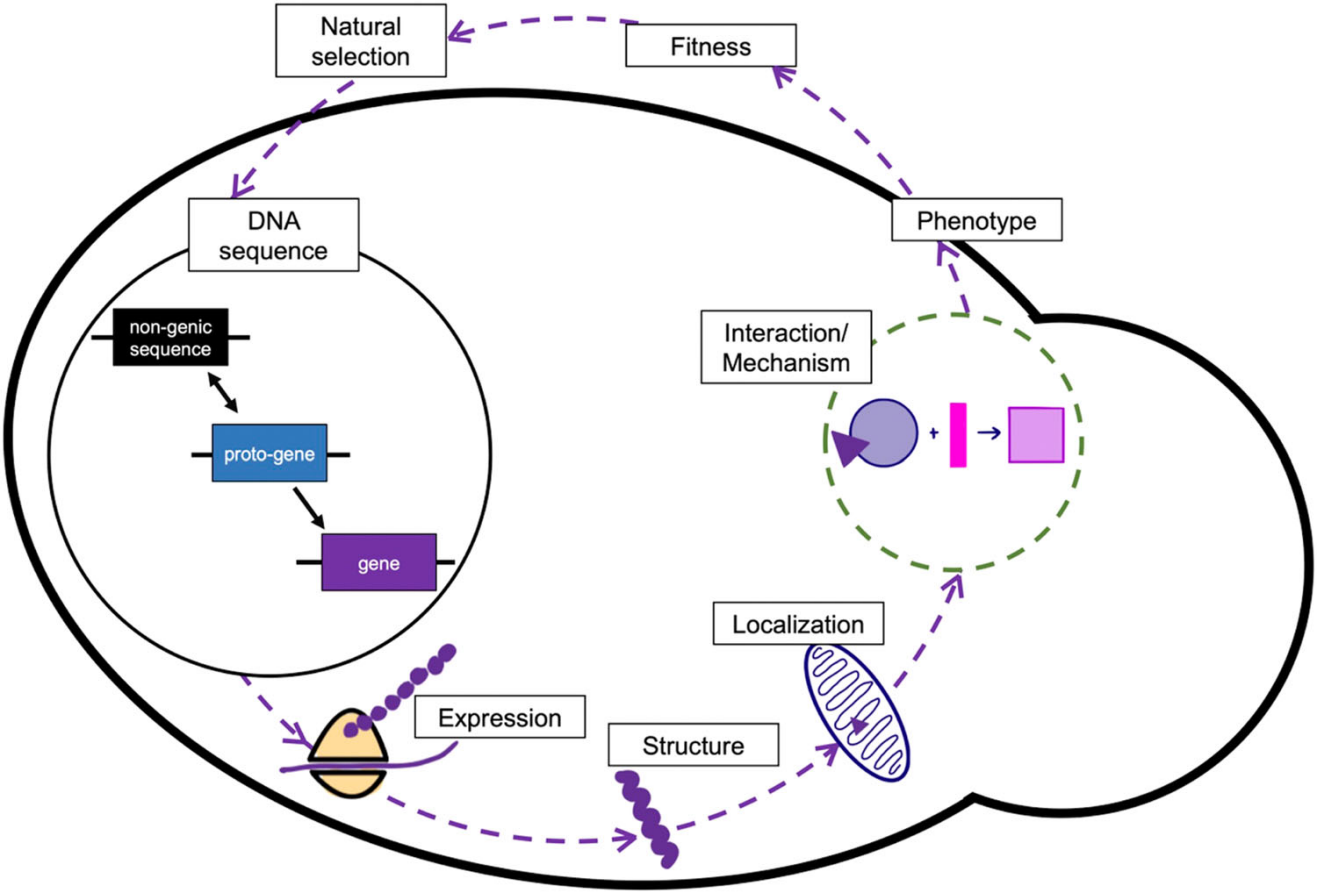
Evolutionary systems biology approach for characterizing the biological role of a candidate de novo gene. The framework proposes a combination of evolutionary and molecular approaches that may be used to identify and investigate a candidate de novo gene. Insights drawn from these varied approaches can then be put together to provide a holistic understanding of the ORF's biology. Overall, this framework represents a circular continuum that is under the influence of natural selection. ORF, open reading frame.

The study of de novo gene birth offers an unprecedented paradigm to understand the role of genetic novelty in the emergence of novel protein structures, functions, and phenotypes. By studying genetic elements that are transitioning from noncoding to protein‐coding, we can unravel how novelty arises on multiple scales, from the DNA sequence to integration into cellular networks and the possible emergence of new phenotypic traits. Given that novel genes, in general, have been shown to rapidly integrate into cellular networks (Abrusan, 2013; Tsai et al., 2012), network‐based approaches may turn out to be very fruitful for understanding what makes a strain or species unique from a molecular standpoint. The example of *MDF1* demonstrates how an emergent de novo protein can rapidly integrate into an existing cellular network and evolve a critical biological role (Figure 1, Table 1) (Li et al., 2010, 2014). For future studies in the emerging field of de novo gene research, we propose a novel framework guided by an evolutionary systems biology approach to utilize yeast's potential as a model and a tool for this field of study (Figure 3). Guided by this framework, related levels of evidence and function (Keeling et al., 2019) can be investigated to characterize de novo ORFs.

For a given de novo candidate, some key questions would be: In what context is it transcribed and translated? When and how did it acquire the regulatory signals controlling expression? Does it participate in genetic or protein–protein interactions? Does it stably localize to a specific subcellular compartment? When and how did its protein sequence acquire the necessary residues or domains to specify its localization and/or interactions? Does its expression impact fitness in a particular biological context? Concomitantly researching a de novo candidate's characteristics along with when and how these characteristics arose is expected to yield insights into the interrelated evolutionary and physiological forces at play. A particular candidate may be required for survival in a specific context, or it may modulate traits that do not impact fitness. As more proto‐genes and de novo genes are discovered, the wealth of resources and the repertoire of techniques available to researchers working in yeast combined with our proposed framework (Figure 3) offer a unique opportunity to explore this untapped font of molecular diversity.

Yeasts are established as an exceptional model for molecular genetics, cell biology, and biochemistry due to their ease of culture, simple life cycles, short generation times, a paucity of multi‐intronic genes, and their relatively small genomes (∼10–20 Mbp.) Genome‐wide deletion and overexpression libraries have been developed for multiple yeast strains, particularly in *S. cerevisiae* (Alberti et al., 2007; Brachmann et al., 1998; Douglas et al., 2012; Fasanello et al., 2020; Gelperin et al., 2005; Giaever et al., 2002; McIsaac et al., 2013; Sopko et al., 2006), enabling advanced, high‐throughput approaches that can be expanded to characterize phenotypes for de novo candidates (Costanzo et al., 2010, 2016; Douglas et al., 2012; Parsons et al., 2006; Piotrowski et al., 2017; Vizeacoumar et al., 2010). Once a phenotype is detected with confidence, mechanisms can be inferred with many tools, for example, with deep mutational scanning (Fowler & Fields, 2014) or network‐based computational approaches (Li et al., 2021). As de novo ORFs often overlap with noncoding sequences that may function as regulatory elements or noncoding RNAs, it can be important to experimentally dissect which aspects of null mutant phenotypes are truly caused by loss of translation or loss of the protein product. This can be achieved with single nucleotide genome editing of the translation start site, for example (Wacholder et al., 2021).

Yeasts have also long been at the forefront of the “omics” revolution, offering the opportunity to conduct systems‐level studies that can investigate the genome, transcriptome, translatome, interactome, metabolome, and phenome (Yu & Nielsen, 2019). Yeast offers yet another advantage over other systems as not only are more and more strains being sequenced every day (Libkind et al., 2020) but such strains are also being used for “comparative‐phenomics” in the laboratory setting (Robinson et al., 2021). The exploitation of natural yeast isolates and diverse experimental conditions that attempt to recreate their natural environment may shed light on why so many de novo translated elements exist, and why they evolve so rapidly. It will be informative to compare the tolerance of de novo ORF expression in wild strains and natural environments with that of commonly used laboratory strains and experimental settings.

Yeasts are also well‐positioned as a model system for addressing the “holy grail” of de novo gene birth: the opportunity to observe the phenomenon in real‐time. While this is not a trivial endeavor, yeasts are amenable to experimental evolution (Voordeckers & Verstrepen, 2015). One can imagine applying selective pressure to an experimentally evolving population and combining it with sequencing to observe the order of events that lead to the formation of de novo genes, and indeed, if a particular path or order is “preferred.” The ability to control this phenomenon opens the possibility that applying appropriate selective pressures may lead to evolved populations with unique genes to overcome current limitations in using yeasts for agricultural, industrial, or medical purposes.

## 6 CONCLUSION

Yeasts are well suited to address some of the fundamental questions and promising opportunities in the de novo gene birth field. The pliability of the system allows us to ask nearly any question: How do de novo ORFs acquire the signals necessary for expression? How are new genetic elements integrated into the vast pre‐existing *S. cerevisiae* transcriptional and protein–protein interaction networks? How can we perturb these networks to dissect the function(s) of these novel genetic elements? The rapidly evolving field of de novo gene birth can shed new light on our understanding of genes, proteins, and how they evolve. Furthermore, it opens the door for exciting medical and industrial possibilities. Yeasts are uniquely situated to exploit these opportunities.

## AUTHOR CONTRIBUTIONS

**Saurin Bipin Parikh and Anne‐Ruxandra Carvunis**: Conceptualization. **Saurin B. Parikh, S. Branden Van Oss, Carly Houghton, Aaron Wacholder**: Writing – original draft. **Saurin B. Parikh, Carly Houghton, S. Branden Van Oss, Aaron Wacholder, Anne‐Ruxandra Carvunis**: Writing – review and editing. **Supervision**: Anne‐Ruxandra Carvunis

## CONFLICT OF INTEREST

Anne‐Ruxandra Carvunis is a member of the scientific advisory board for Flagship Labs 69 Inc. (ProFound Therapeutics).
